# Differential Mortality Rates by Ethnicity in 3 Influenza Pandemics Over a Century, New Zealand

**DOI:** 10.3201/eid1801.110035

**Published:** 2012-01

**Authors:** Nick Wilson, Lucy Telfar Barnard, Jennifer A. Summers, G. Dennis Shanks, Michael G. Baker

**Affiliations:** University of Otago, Wellington, New Zealand (N. Wilson, L. Telfar Barnard, J.A. Summers, M.G. Baker);; Australian Army Malaria Institute, Enoggera, Queensland, Australia (G.D. Shanks)

**Keywords:** influenza pandemic, virus, historical, Maori, Pacific

## Abstract

Evidence suggests that indigenous populations have suffered disproportionately from past influenza pandemics. To examine any such patterns for Māori in New Zealand, we searched the literature and performed new analyses by using additional datasets. The Māori death rate in the 1918 pandemic (4,230/100,000 population) was 7.3× the European rate. In the 1957 pandemic, the Māori death rate (40/100,000) was 6.2× the European rate. In the 2009 pandemic, the Māori rate was higher than the European rate (rate ratio 2.6, 95% confidence interval 1.3–5.3). These findings suggest some decline in pandemic-related ethnic inequalities in death rates over the past century. Nevertheless, the persistent excess in adverse outcomes for Māori, and for Pacific persons residing in New Zealand, highlights the need for improved public health responses.

## Abstract 2

The persistent excess in adverse outcomes by ethnicity highlights the need for improved public health responses.

Evidence suggests that indigenous populations have been disproportionately affected more by influenza pandemics than other population groups. In the most detailed review to date for the Spanish influenza pandemic (1918–1920), Mamelund (*1*) reported elevated mortality rate ratios (RRs) for indigenous populations relative to European populations in North America: the continental United States (RR 3.2); Alaska (RR range 6.8–191.5); all of Canada (RR 4.8); Labrador, Canada (RR range 8.3–129.0) and Greenland (RR 4.9). This pattern was also apparent for the Sami in Nordic countries of Norway (RR 4.8), Sweden (RR 8.2), and Finland (RR 16.9). Indigenous Australians were particularly affected (RR 172.4), but so were indigenous Pacific persons in Guam (RR 3.2), Fiji (RR 4.8), Tonga (RR range 2.6–5.3), Samoa (RR 16.5), Nauru (RR 11.2), Tahiti (RR 10.9), and Hawaii (RR 4.1).

In contrast, little is known about ethnic gradients in outcomes for other influenza pandemics of the 20th century, such as the 1957 pandemic. More recently, many studies have considered the 2009 influenza pandemic, and there are reports of increased risk for either hospitalization or death for indigenous persons from Canada, the United States, Brazil, Australia, New Zealand, and New Caledonia (*2*,*3*). Other work involving 12 US states indicated elevated mortality RRs for American Indian/Alaska Natives (RR 4.0, 95% confidence interval [CI] 2.9–5.6) (*4*). Canadian research also identified First Nations (indigenous) ethnicity as an independent risk factor for increased disease severity, with the multivariable model accounting for age, sex, medical comorbidity, interval from onset of symptoms to initiation of antiviral therapy, rurality, and income (*5*). That is, First Nations ethnicity was associated with increased likelihood of being admitted to an intensive care unit (odds ratio 6.52, 95% CI 2.04–20.8), but this pattern was not seen for low-income persons or those residing in rural areas.

Despite this historical and more recent work, little evidence exists concerning how the ethnic mortality differential of pandemic influenza may have changed over time. Therefore, in this study we considered such data for Māori (the indigenous population of New Zealand) and to some extent for Pacific populations residing in this country.

## Methods

We searched the literature (Medline and Google Scholar) for relevant publications relating to New Zealand up to December 1, 2010. Search terms used were combinations of influenza and Māori/Pacific and New Zealand. The bibliography of a key text (*6*) was used to help identify this literature.

### 1918–19 Pandemic

**Figure 1:**
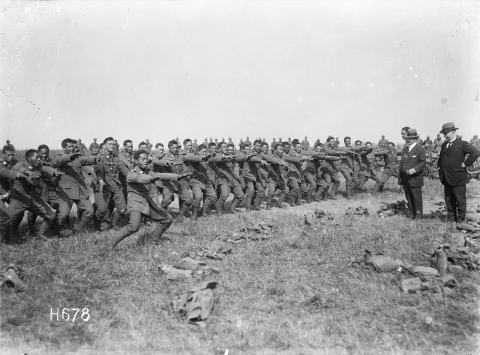
Māori military personnel (Pioneer Battalion) performing the haka for New Zealand Cabinet Minister Sir Joseph Ward (at Bois de Warnimont, France, June 30, 1918). Photograph taken by Henry Armytage Sanders; from Alexander Turnbull Library, Timeframes: New Zealand and the Pacific through images; reference no. 1/2-013283-G (http://timeframes.natlib.govt.nz).

Mortality rate data for military personnel in the New Zealand Expeditionary Force (NZEF) were obtained from an electronic dataset (Roll-of-Honor) covering all deaths in these personnel during World War I (WWI) (Figure 1). This dataset was obtained courtesy of the compiler, Professor Peter Dennis (University of New South Wales at the Australian Defence Force Academy). Those reported as dying of disease or from no specified cause were studied further, and if dying during known pandemic periods, they were considered to have died from pandemic influenza (i.e., after excluding deaths that had a specific alternative diagnosis in the archival records). These pandemic periods were 1) August 27–December 31, 1918, for the second wave in the Northern Hemisphere); 2) January 1–March 31, 1919, for the third wave in the Northern Hemisphere; and 3) November 1–December 31, 1918, for the second wave in the Southern Hemisphere. There was no evidence for excess mortality rates in military personnel from any first or third pandemic wave in the Southern Hemisphere.

Military personnel were classified as having Māori ethnicity if they: had a first, second, or surname in the Māori language; had a parent with a Māori language name; were buried in a Māori cemetery or had a memorial in such a cemetery; or had a iwi (tribal) affiliation listed in the Cenotaph database covering NZEF personnel (purchased from the Auckland War Memorial Museum [*7*]). Personnel were classified as having Pacific person ethnicity if they came from a South Pacific island providing military personnel for the NZEF (i.e., Fiji, Gilbert and Ellis Islands, Niue, Samoa, Tonga, and the Cook Islands) and had a Pacific name or a parent with a Pacific name or came from a named village. The approach of using the language of a name for considering ethnicity has been used elsewhere in historical work in New Zealand (*8*). It is also used for identifying from the electoral roll potential Pacific persons as respondents to public health surveys in New Zealand (the Lexicon method used by Massey University) (*9*).

For denominator data, we extracted a random sample of 1,000 persons (≈1%) who served in the NZEF as detailed in the Cenotaph database. This denominator sample was then adjusted further to replace (with additional random selection) those who died in the prepandemic period. Ethnicity coding was then performed as for the numerator data.

A validation study was performed for the method of ethnicity coding for Māori; this study involved a University of Otago colleague with local history expertise (Dr George Thomson) who independently classified the ethnicity of WWI participants in a rural area in which he had performed historical research. The results indicated that the coding system we have used was underascertaining Māori ethnicity (sensitivity 73%, i.e., n = 11/15). Of note, however, is that the rural locality used in this validation study had a relatively high Māori population in the pre-WWI era, and intermarriage between Māori and New Zealand European persons was relatively common. As such, the underascertainment found would be a worst-case assessment if applied to New Zealand in general. In contrast, all of those classified as Māori by our coding system were also classified as Māori by Dr Thomson (specificity 100%, n = 17/17).

### 1957 Pandemic

In addition to considering Māori mortality rates in official data identified in our literature search, we undertook an additional analysis of an online national database for deaths in New Zealand (*10*). Because ethnicity coding at this time in New Zealand history was poor, we again adopted the approach of using the language of the surname. We examined surnames in the Māori language by using the most common 50 surnames in the 2006 Māori electoral roll. Similarly, this examination was performed for the most common 10 surnames from the general electoral roll of 2006 (all of these were surnames of European origin). The periods considered were for August–September in 1956, 1957, and 1958; each of these months had maximum pandemic impact in 1957 (*11*). The 1956 and 1958 periods were used for comparison with the known pandemic period in 1957. This method was also used to identify the increased mortality rate for the 1890s pandemic for the European population because registration of Māori deaths was not routine in the 19th century. We did not study the Hong Kong influenza pandemic of 1968–1969 because we could find no published studies in New Zealand and no evidence for increased mortality rates in the annual mortality data (*12*).

A limitation with this surname method was that the number of Māori deaths involved was small, e.g., 23 and 28 deaths in August–September in 1956 and 1958 respectively, compared with 38 deaths in August–September in 1957 (using the top 50 surnames in Māori language). Furthermore, some persons with Māori surnames may not have been Māori (e.g., non-Māori women who married Māori men with Māori surnames). Also, a proportion of the total New Zealand mortality rate would have included Māori deaths because the most common 7 surnames on the Māori electoral roll (2006) are actually names of European origin that Māori have commonly adopted over the past 150 years (e.g., Smith, Williams, Brown, Wilson; Kingi, the first name in te reo Māori, is listed eighth). The latter factor is likely to dominate. Thus, our analysis is likely to have underestimated the true pandemic-related Māori mortality rate.

### 2009 Pandemic

Anonymized mortality data were obtained from an official mortality review group that was charged by the New Zealand government with identifying deaths caused by pandemic (H1N1) 2009 (*13*). All but 1 of the 49 deaths included in our analysis occurred during June 1–September 30, 2009. The review group used prioritized ethnicity (i.e., where there are multiple ethnicities assigned, Māori ethnicity takes precedence, followed by Pacific persons, with the remaining persons included as European and others). Rates and RRs were calculated and age-standardized to the 2006 New Zealand Census with Māori as the standard population (the Māori population has a younger age structure than the European population and is more similar to Segi’s world standard population).

## Results

### 1918–19 Pandemic

**Figure 2:**
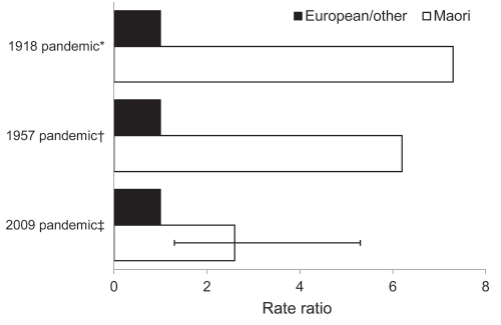
Mortality rate ratios (age-standardized on the basis of 2009 data) for Māori versus European/other New Zealanders (non-Māori/non-Pacific) during 3 influenza pandemics in New Zealand. *Data from (*6*); †official mortality rate data; ‡age-standardized to the Māori population. Error bar represents 95% CI.

During the 1918 pandemic, the Māori mortality rate (4,230/100,000 population) was 7.3× the rate for the rest of the population (*6*), which was nearly all composed of settlers of European origin (Table; Figure 2). This work by Rice (*14*) was far more comprehensive than previous work that compared rates by ethnicity (*15*). Nevertheless, Rice noted limitations with data quality and considered that the Māori rate was still an underestimate of the true rate because some Māori deaths were not registered (*14*). This underestimation of Māori pandemic-related deaths was also a finding in a study of the pandemic in New Zealand’s largest city, Auckland (*16*).

**Table:** Comparison of mortality rates for Māori versus non-Māori/European residents of New Zealand during multiple influenza pandemics*

Pandemic and data source	Mortality rate			Comments (see Methods for details)
	Māori	Non-Māori	Ratio†	
1890s pandemic				
Individual mortality data in BDM database (*10*)	Unknown (deaths not registered)	9.1% increase in deaths for 1890–94 compared with 1885–89	–	Based on comparison of no. deaths for top 10 surnames (see method used for 1957 pandemic). Official data also suggest increased influenza deaths for the 1890s beginning in 1890 (*17*).
1918–19 pandemic				
National mortality data, second wave (*6*), n = 8,573 deaths	4,230/100,000 population	580/100,000 population) (European)	7.3	See limitations with data quality described in the main text. Comparison was not age-standardized.
Mortality in New Zealand military personnel, second and third waves,‡ n = 1,113	2,501/100,000 population	1,103/100,000 population (European/ other)	2.3 (1.6–3.1)§	New Zealand military personnel of Pacific peoples ethnicity also had a raised mortality rate, but absolute number of deaths was small (n = 12) and difference was not significant.
1957 pandemic				
National mortality data for Asian influenza pandemic, official report (*11*), n = 179 deaths	39.6/100,000 population	6.4 per 100,000 population (European)	6.2	Of note, at this time surveillance systems were crude, and attention to quality ethnicity coding was not robust. There was no widespread use of vaccination in response to this pandemic in New Zealand.
Individual mortality data in BDM database for selected surnames (*10*), n = 38 deaths in Aug/Sep 1957	49.0% increase for Aug/Sep 1957 compared with same period in 1956 and 1958	Whole New Zealand population: 20.3% increase	2.4 (Māori vs. total population)	See Discussion for limitations with this method.
2009 pandemic¶				
All cases with pandemic (H1N1) 2009 as primary cause of death,# n = 49	2.0 (0.8–3.1)§	0.8 (0.5–1.1)§	2.6 (1.3–5.3)§	For Pacific peoples in New Zealand, rate = 4.6 (2.0–7.2)§

*BDM, Births, Deaths & Marriages. †Māori:non-Māori except as indicated. ‡Age standardization was not possible with available data; 1 study reported that Māori and European soldiers had similar median ages of 24 and 26 years, respectively (*8*). §Values in parentheses are 95% confidence intervals. ¶Jun–Sep 2009. Cumulative age-standardized mortality rates per 100,000 population, age-standardized to Māori population. Ratio is Māori versus other New Zealanders (non-Māori and non-Pacific, mainly European). #Identified by Mortality Review Group.

The analysis of the effect of 2 pandemic waves among New Zealand military personnel found a substantially higher mortality rate among Māori personnel (2,501/100,000) than for European military personnel (Table). The rate for Māori soldiers was 2.3× higher than that for European soldiers.

### 1957 Pandemic

In this pandemic, the Māori mortality rate (39.6/100,000) was 6.2× the European rate (Table; Figure 2). In our new analysis using selected surnames, an equivalent ratio 2.4× higher for Māori was found for 2 months when the pandemic was most active in New Zealand in 1957 (Table).

### 2009 Pandemic

A national serosurvey after wave 1 of the pandemic found that Māori had evidence of higher seroprevalence of antibodies to the pandemic (H1N1) 2009 virus but not at statistically significant levels (Māori 36.3%, 95% CI 28.0–44.6, vs. other [mainly European] 25.9%, 95% CI 22.4–29.4) (*18*). However, multivariate analysis identified significantly higher seroprevalence rates for Pacific populations (49.5%; adjusted odds ratio 2.2, 95% CI 1.5–3.4) compared with the other ethnic group population (i.e., mainly European New Zealanders).

### 2009 Pandemic Outcomes

During the 2009 pandemic, Māori had relatively higher notification rates for pandemic influenza (age-standardized relative risk [aRR] 2.0, 95% CI 1.9–2.1) compared with Europeans and others (and similarly for Pacific persons, aRR 4.0, 95% CI 3.8–4.3) (*19*). The cumulative age-standardized hospitalization rate for Māori was 43.0/100,000 compared with Europeans and others at 14.1/100,000 (Māori aRR 3.0, 95% CI 2.9–3.2; Pacific persons aRR 6.7, 95% CI 6.2–7.1) (*19*). A local study also found higher hospitalization rates for Māori in the Wellington region, at 5× the rate for other ethnic groups (excluding Pacific persons) (*20*).

Intensive care unit (ICU) admissions were also significantly higher for Māori and Pacific persons compared with Europeans (*13*). More specifically, Māori and Pacific women who were pregnant were more likely to have an ICU admission compared with other pregnant women (RR 2.3, 95% CI 1.4–3.7) (*21*).

Our analysis of national mortality data found that the Māori rate was significantly higher (2.6×) than the rate for other New Zealanders (non-Māori and non-Pacific New Zealanders, largely European) (Table). Similarly, for Pacific persons, the rate was 5.8× higher than that for other New Zealanders (Table). An official post-pandemic review also reported elevated rates for both ethnic groups, but it did not perform age standardization of rates (*13*).

### Risk Factors (All Pandemics)

None of the work on these 3 pandemics systematically analyzed risk factors for adverse outcomes of pandemic influenza on Māori. Nevertheless, in 1918 the high rate of illness and death among adult caregivers is thought to have limited capacity to provide basic care for others, contributing to relatively high total Māori mortality overall (*6*,*15*). Furthermore, various supportive care services in towns and cities may have favored better health outcomes for the affected European population relative to Māori (*6*). Māori were largely rurally based in 1918 (*22*) and rural health services were limited at that time.

For the 2009 pandemic, it was reported that 86% of those who died had >1 concurrent or associated condition, particularly obesity (74%), morbid obesity (56%), and respiratory disease (49%) (*13*). Furthermore, 39% of those who died had a deprivation score of either 9 or 10 (most deprived) compared with the expected 20% of the population (using a New Zealand–specific small area deprivation index). Similarly, the ICU study identified risk factors for pandemic influenza–related admission, including pregnancy, body mass index >35, and preexisting asthma or other chronic pulmonary disease (*23*).

## Discussion

The mortality rates from pandemic influenza for Māori and European New Zealanders declined markedly over the 3 pandemics. Nevertheless, this finding may only partly represent improved public health controls and health care, given marked variation in virulence of difference pandemic influenza viruses over this period.

In terms of relative inequalities, the excess in Māori mortality rates (compared with those of European New Zealanders) appears to have declined over the 3 pandemics. However, the persistently poorer health outcomes of Māori during the 2009 pandemic is of continuing concern and is compatible with other evidence for persisting inequalities in major health risk factors (*24*) and in health outcomes when comparing Māori with non-Māori (*25*). In particular, total Māori hospitalization rates for infectious diseases have been consistently double those seen for the European/other population over the 20-year period of 1989–2008 (*26*). Māori also have markedly elevated rates for several specific infectious diseases for which household crowding has been a risk factor in the New Zealand setting, i.e., tuberculosis (*27*), meningococcal disease (*28*), and rheumatic fever (*29*).

Other possible factors for poorer pandemic influenza outcomes in indigenous populations (*2*) include 1) a higher prevalence of infection (but this was not found to be significant in the New Zealand seroprevalence study); 2) higher prevalence of concurrent conditions (which is relevant for Māori, particularly in terms of diabetes and chronic respiratory diseases [*25*]); and 3) possibly poorer access to health services. Differential use of seasonal influenza vaccination by ethnic group affiliation might also be relevant, but the literature regarding the benefit of prepandemic seasonal vaccination for pandemic (H1N1) 2009 outcomes remains unclear (*18*).

The results for Māori are compatible with the findings for Pacific persons serving in the military in 1918 and living in New Zealand in 2009 (Table). Like Māori, these persons are largely of Polynesian ethnicity and are less socioeconomically advantaged relative to the European New Zealander ethnic group. Furthermore, for other Pacific persons such as those living in New Caledonia, there appear to have been increased adverse outcomes from the 2009 pandemic (*2*,*30*).

The relatively higher mortality rate from pandemic influenza for Māori military personnel in 1918 indicates that the excess pandemic effect was even seen among relatively fit young men. One possible explanation for this pattern comes from a large study of Australian soldiers in 1918, which suggests that one’s previous experience with respiratory pathogens was an important protective factor in the pre–antimicrobial drug era (*31*). Preexisting immunity was also a probable important risk factor in a study of 1918 mortality among the Sami of Norway in a multivariate analysis that considered such factors as wealth, poverty, crowding, and occupational structure (*32*). Other similar work, but on an international scale, has suggested that geographic remoteness (associated with less frequent exposure to other forms of influenza) may have contributed to high mortality rates in indigenous persons (*1*). This work also suggested plausible roles for high concurrent disease load, crowding, and a lack of basic care (i.e., for dependents when many younger adults died suddenly).

Concurrent disease incidence is a plausible risk factor in the 1918 pandemic for Māori soldiers and the civilians in 1918, given the much higher rates of other infectious diseases such as tuberculosis (*33*). Relative poor nutritional status may also have mattered for the civilian population of Māori, but for the military population no evidence (*34*,*35*) of any poorer provision of supplies for Māori soldiers compared with European soldiers was found (and similarly for the quality of accommodation or health care services). Also, 1 study reported no differences in stature and only small differences in median weight for Māori compared with European military recruits during WWI (160 vs. 150 lbs., respectively) (*8*). Little information is available on the 1957 pandemic, but concurrent conditions, e.g., higher tuberculosis rates (*33*), poorer housing, and poorer access to health services, are plausible risk factors.

New Zealand data made it possible to describe health outcomes by ethnicity for pandemics from 1918 onwards. Integrated national-level data collection systems for notifications, hospitalizations and deaths also facilitated such comparisons for the 2009 pandemic.

A limitation of the 1918 data identified was the incomplete ascertainment of pandemic-related deaths among Māori, caused partly by incomplete death registrations in this population (*6*,*16*). Similarly, a limitation with our analysis of the military personnel for 1918–19 was that the ethnicity classification system used is likely to have underestimated Māori.

Māori deaths occurring during 1957 may have also been underestimated because ethnicity classification at this time was based on funeral director assessments. We acknowledge the limitations of using the language of surnames as part of ethnicity coding and the likelihood of underestimating the mortality rates for Māori; practical alternatives for analyzing such historical data are lacking.

To identify risk factors for these ethnic disparities, analytic epidemiologic methods are needed, such as case–control studies of the 1918 military personnel for which there are detailed archival data and hospitalization records for 2009. Such studies need to include risk factors for influenza infection as well as possible risk factors for adverse outcomes, e.g., low socioeconomic position, household crowding, smoking, obesity, preexisting chronic diseases, the lack of prior seasonal influenza vaccination or pneumococcal vaccination, and poorer access to healthcare. Indeed, further detailed work is underway on the ethnic gradient for influenza for 2009 in New Zealand by some of us with Māori health colleagues.

Although further research is desirable, enough is now known about health inequalities in nations with indigenous peoples for government agencies and health care workers to pursue specific interventions. For example, in New Zealand, interventions should continue to raise the social and economic well-being of Māori, i.e., improve housing, reduce smoking, control obesity, improve management of diabetes, increase immunization rates, and improve access to health care services. Fortunately, many such interventions are part of New Zealand health sector activity and range from national-level smoking cessation campaigns to more local community-level programs such as a Let’s Beat Diabetes program. Other policy development is occurring, with new and substantive tobacco control measures recommended by a Māori Affairs Select Committee in late 2010 (*36*). These responses are also relevant to improving health protection for Pacific persons, albeit in different ways that are also culturally appropriate. In addition, improvements are needed for influenza vaccination coverage in more vulnerable populations and more pandemic planning on how to reduce inequalities in influenza outcomes for indigenous persons (highlighted in work to protect Australian Aboriginal peoples [*37**,**38*]; indigenous persons in the United States [*39*], and other ethnic groups in the United States [*40*]).

Analysis of multiple health data sources indicates large reductions in absolute mortality rates from pandemic influenza for Māori and European New Zealanders and is suggestive of some decline in relative ethnic health inequalities for pandemic mortality over the past century. However, the persistent Māori excess in hospitalizations and deaths for the 2009 pandemic highlights the need for additional research to clarify contributing factors. There remains an ongoing need for societal and public health action to reduce known risk factors for influenza infection and adverse health outcomes for indigenous populations such as Māori.
